# The extrahepatic role of TFR2 in iron homeostasis

**DOI:** 10.3389/fphar.2014.00093

**Published:** 2014-05-07

**Authors:** Laura Silvestri, Antonella Nai, Alessia Pagani, Clara Camaschella

**Affiliations:** Division of Genetics and Cell Biology, San Raffaele Scientific Institute, Università Vita-Salute San RaffaeleMilan, Italy

**Keywords:** iron metabolism, transferrin, transferrin receptors, hepcidin, iron deficiency, hemochromatosis

## Abstract

Transferrin receptor 2 (TFR2), a protein homologous to the cell iron importer TFR1, is expressed in the liver and erythroid cells and is reported to bind diferric transferrin, although at lower affinity than TFR1. *TFR2* gene is mutated in type 3 hemochromatosis, a disorder characterized by iron overload and inability to upregulate hepcidin in response to iron. Liver TFR2 is considered a sensor of diferric transferrin, possibly in a complex with hemochromatosis protein. In erythroid cells TFR2 is a partner of erythropoietin receptor (EPOR) and stabilizes the receptor on the cell surface. However, *Tfr2* null mice as well as *TFR2* hemochromatosis patients do not show defective erythropoiesis and tolerate repeated phlebotomy. The iron deficient *Tfr2-Tmprss6* double knock out mice have higher red cells count and more severe microcytosis than the liver-specific *Tfr2* and *Tmprss6* double knock out mice. TFR2 in the bone marrow might be a sensor of iron deficiency that protects against excessive microcytosis in a way that involves EPOR, although the mechanisms remain to be worked out.

## THE SECOND TRANSFERRIN RECEPTOR

Transferrin receptor 2 (TFR2) is a type II transmembrane glycoprotein, member of the TFR family and homologous to TFR1, which provides iron to the cell by internalization of the transferrin-iron complex through receptor-mediated endocytosis. TFR2 was cloned during a project aimed at isolating genes encoding new transcription factors (Kawabata et al., 1999). The *TFR2* gene comprises 18 exons and maps to chromosome 7q22 in close proximity to the EPO gene. Two *TFR2* isoforms have been described: the alpha isoform, corresponding to all 18 exons, encodes a protein of about 89 kDa in its unglycosylated form. The beta form, which results from an alternative splicing, lacks exons 1–3, and has 142 additional nucleotides in its first exon (exon 4 of the alpha form). The resulting protein lacks the cytoplasmic and the transmembrane domain (Kawabata et al., 1999) and its function remains unknown. The alpha protein encompasses 801 amino acids and, as TFR1, has a short cytoplasmic tail that contains a consensus sequence for endocytosis, a transmembrane domain and a large extracellular region that comprises a protease-associated domain and two RGD motifs (only one is present in TFR1), that bind diferric transferrin. TFR2 differs from TFR1 for several aspects. First, TFR1 is ubiquitously expressed, whereas the expression of TFR2 is restricted to the hepatocytes and erythroid precursors. Second, TFR1 is post-transcriptionally regulated by iron through the iron regulatory proteins–iron responsive elements (IRP–IRE) interaction, while TFR2 is not regulated by IRPs. TFR2 5′ and 3′ untranslated regions do not contain IRE elements (Fleming et al., 2000); rather TFR2 promoter shows GATA-1 as well as c-EBP-alpha consensus sequences. Furthermore, although in transfected cells TFR2 may uptake iron loaded transferrin (holo-TF) in transfected cells, *in vivo* it does not rescue the embryo-lethality of *Tfr1*^-/-^ mice (Levy et al., 1999), suggesting a function distinct from TFR1 and unrelated to iron transport. Moreover, the affinity of TFR2 for holo-TF is significantly lower than that of TFR1 (Kd 30 nM vs. 1 nM, respectively; Kawabata et al., 2000; West et al., 2000).

It has been reported that both TFRs bind hemochromatosis protein (HFE) *in vitro*. However, crystallographic studies have shown that HFE binds TFR1 (Bennett et al., 2000) at the same consensus sequences of diferric transferrin, implying a competition between the two ligands. On the contrary, based on *in vitro* data, binding of HFE to TFR2 and holo-Tf would occur simultaneously at two different TFR2 sequence motifs (Gao et al., 2009).

In the liver *Tfr2* expression increases during mouse development, at variance with *Tfr1*, and in adult liver *Tfr2* is much more expressed than *Tfr1* (Kawabata et al., 2001). Our knowledge of the TFR2 hepatic function is still incomplete. In hepatoma cell lines TFR2 is stabilized on cell surface by the addition of holo-transferrin to the culture media, an effect due to the increased protein half-life (Enns, 2001). The divergent iron-mediated regulation of the two TFRs is confirmed also *in vivo*: while in iron loaded mice *Tfr1* is downregulated by the loss of IRP-mediated mRNA stabilization, Tfr2 protein level is increased. In agreement with a ligand-mediated stabilization, levels of Tfr2 protein are decreased in the liver of hypotransferrinemic (*hpx*) mice (Robb and Wessling-Resnick, 2004). Thus the major regulation of TFR2 occurs at the protein rather than at RNA level.

In cell culture models, TFR2 localizes in caveolar microdomains (Calzolari et al., 2006), membrane structures involved in the recruitment of receptors that can be activated by ligand binding (Simons and Toomre, 2000). In the absence of holo-transferrin, both TFR1 and TFR2 are internalized by clathrin-mediated endocytosis (Chen et al., 2009), whereas in the presence of the ligand only TFR2, and not TFR1, activates ERK1/2 and p38 MAPK. This has been observed in hepatoma derived and in erythroid cells, supporting the hypothesis that TFR2 may function as a signaling receptor (Calzolari et al., 2006; Poli et al., 2010).

The stabilization of TFR2 by holo-transferrin and its ability to bind HFE led to the current model in which liver TFR2, in conjunction with HFE, represents a sensor of circulating iron and activates hepcidin in response to elevated transferrin saturation (Goswami and Andrews, 2006). In addition it has been shown that the TFR2-HFE interaction on the hepatocyte surface occurs within a multiprotein complex, that *in vitro* includes also the BMP-coreceptor hemojuvelin (D’Alessio et al., 2012). If this complex activates the intracellular signaling to upregulate hepcidin expression *in vivo* remains to be demonstrated. In addition, the binding of TFR2 to HFE has recently been questioned (Rishi et al., 2013) and some evidences are in favor of a distinct function for the two HFEs. Mice with inactivation of both *Tfr2* and *Hfe* have a more severe phenotype compared to single mutant animals (Wallace et al., 2009). This occurs also in humans: patients with mutation in *TFR2* and *HFE* were reported to develop a severe form of juvenile-like hemochromatosis (Pietrangelo et al., 2005). Moreover, patients with *TFR2* mutations do not upregulate hepcidin upon oral iron administration, whereas the iron response is partially preserved in *HFE* patients (Girelli et al., 2011), thus strengthening the distinct and non-overlapping role of HFE and TFR2.

## TFR2: THE GENE OF HEMOCHROMATOSIS TYPE 3

In humans inactivating mutations of TFR2 lead to hemochromatosis type 3 (Camaschella et al., 2000), a rare recessive disorder characterized by iron overload, low hepcidin levels (Nemeth et al., 2005) and inability to properly regulate hepcidin after an oral iron challenge (Girelli et al., 2011). The disorder is quite rare among Caucasians and occasionally reported in Japanese, with single families identified in France, Portugal Spain and Taiwan. Currently, less than 30 pathogenic mutations have been described (Camaschella and Roetto, 2011). They are all rare, often private. Some insertions cause frameshift and premature stop codon, others are nonsense and small deletions. All the mutations are loss of function; missense mutations prevalently affect the protein C-terminus, especially the peptidase-like and the dimerization domains, suggesting that these regions have important functional roles (Camaschella and Roetto, 2011). The affected residues, usually highly conserved, might be essential for the proper folding and protein localization, or relevant for interaction with other proteins or for the regulatory function of TFR2.

The type of iron overload caused by mutations in TFR2 differs from the classic type 1 HFE-hemochromatosis, because of an earlier onset and more severe presentation (Camaschella, 2005). A study in patients affected by type 1 (HFE-related) or type 3 (TFR2-related) hemochromatosis showed a different role for TFR2 and HFE in hepcidin activation in response to a single oral iron challenge able to increase transferrin saturation: HFE patients showed a blunted hepcidin response, whereas TFR2 patients showed no response (Girelli et al., 2011). A similar difference in the hepcidin response after an acute iron loading has been observed in *Tfr2*^Y245X/Y245X^ and *Hfe*^-/-^ mice (Corradini et al., 2011; Ramos et al., 2011). These results led to the conclusion that TFR2 is important to up-regulate hepcidin in response to transferrin saturation.

## TFR2 IN ERYTHROID CELLS

In favor of the relevance of TFR2 for the erythroid differentiation are two genetic observations: first, the close proximity of TFR2 and EPO genes on chromosome 7q22 that may suggest a common regulation. Second, the results of different genome-wide association studies: TFR2 single nucleotide polymorphisms (SNPs) have been identified associated with erythroid quantitative traits, such as red cell numbers, indexes and hematocrit (Ganesh et al., 2009; Soranzo et al., 2009; Ding et al., 2012). Although this observation could be due to an indirect effect mediated by serum iron levels, that were not measured in the original studies (Ganesh et al., 2009; Soranzo et al., 2009; Ding et al., 2012), a direct effect of TFR2 on erythropoiesis cannot be excluded.

More recently it was shown that in erythroid cells TFR2 is a partner of erythropoietin receptor (EPOR) that stabilizes the receptor on cell surface. *TFR2* is co-expressed with *EPOR* during erythroid differentiation and its maximal expression precedes that of *TFR1* (Kawabata et al., 2001). TFR2 protein, that associates with EPOR in the endoplasmic reticulum, is needed for the efficient transport of the receptor to the cell membrane (Forejtnikova et al., 2010). The interaction facilitates the stabilization of EPOR, likely contributing to EPO sensitivity and erythroid cell differentiation, both *in vitro* and *in vivo*. However the interaction does not seem to influence EPO binding to EPOR. In addition direct binding of TFR2 to EPO was excluded (Forejtnikova et al., 2010). Interestingly *TFR2* silencing in human erythroid precursors delays their terminal differentiation *in vitro* (Forejtnikova et al., 2010). However, which signaling pathway is activated by TFR2 is still unclear. Moreover, although TFR2 is required for efficient erythropoiesis, *Tfr2* null mice as well as *TFR2* hemochromatosis patients do not show defective erythropoiesis and patients tolerate repeated courses of phlebotomy without developing anemia.

## ANIMAL MODELS OF *Tfr*2 INACTIVATION DEVELOP IRON OVERLOAD

The first animal model of hemochromatosis type 3 was generated by targeted mutagenesis, introducing a premature stop codon (Y245X; Fleming et al., 2002) in the murine *Tfr2* coding sequence. This mutation is orthologous to the mutation (Y250X) originally detected in humans (Camaschella et al., 2000). Young (4 week-old) homozygous Y245X mutant mice had high liver iron concentration, even if maintained on a standard diet, in agreement with the observation of early iron overload in patients. The histological distribution of iron recapitulates features of hemochromatosis, with the typical liver periportal accumulation and low spleen iron stores. As in humans, heterozygous animals were normal. Later on, several murine models of *Tfr2* inactivation were developed (Fleming et al., 2002; Wallace et al., 2007), including, among others, *Tfr2* total (*Tfr2*^-/-^) and liver-specific (*Tfr2* ^LCKO^) knock-out (Wallace et al., 2007; Roetto et al., 2010) and *Tfr2-Hfe* double knock-out. All these models are characterized by low hepcidin expression and liver iron overload of variable severity (**Table 1**). However, when generated in the same genetic background, *Tfr2* total knock-out was shown to have iron overload more severe than *Hfe*^-/-^ although less severe than *Hfe/Tfr2* double knock out. These observations are in agreement with the suggested distinct function of the two proteins.

**Table 1:** Hepcidin levels, iron and hematological phenotype in the available murine models of *Tfr2* inactivation.

Mouse model	Hepcidin expression	Iron phenotype	Hematological phenotype		Reference
			RBC	Hb	
*Tfr2^Y245X/Y245X^*	Reduced^*^	Iron overload	n.a	Normal	Fleming et al. (2002), Kawabata et al. (2005)
*Tfr2*^-/-^	Reduced^*^	Iron overload	Normal	High	Roetto et al. (2010), Wallace et al. (2005)
*Tfr2^LCKO^*	Reduced^*^	Severe iron overload	Normal	Normal	Roetto et al. (2010), Wallace et al. (2007)
*Tfr2^KI^*	Normal	Moderate iron overload (old mice)	Low	Low	Roetto et al. (2010)
			(only in young mice)		
*Tfr2*^-/-^*Hfe*^-/-^	Low	Severe iron overload	n.a	n.a.	Wallace et al. (2009)
*Tfr2^Y245X/Y245X^Hfe*^-/-^	Very low	Severe iron overload	n.a	n.a.	Corradini et al. (2011)
*Tfr2^Y245X/Y245X^Tmprss6^msk/msk^*	High	Iron deficiency	High	Low	Lee et al. (2012)
*Tfr2*^-/-^*Tmprss6*^-/-^	High	Iron deficiency	High	Low	Nai et al. (2014)
*Tfr2^LCKO^Tmprss6*^-/-^	Very high	Iron deficiency	High	Low	Nai et al. (2014)

*RBC, red blood cells; Hb, hemoglobin.*

* Reduced compared to the level of iron-loaded mice; n.a = not available.

A last model was generated with the M167K substitution in the Tfr2 protein (Roetto et al., 2010): this mutation destroys the methionine, putative start codon of the beta-isoform of the protein. β-Tfr2 is mostly expressed in the spleen (Kawabata et al., 1999; Roetto et al., 2010). Interestingly, the knock-in model *Tfr2*^KI^, specifically lacking the beta-isoform, is characterized by normal transferrin saturation, liver iron concentration, hepcidin and *Bmp6* levels but show a transient anemia at young age. In addition adult animals accumulate iron in the spleen due to strong reduction of ferroportin mRNA, thus suggesting a possible regulatory effect of β-Tfr2 on splenic ferroportin expression.

## ANIMAL MODELS OF *Tfr*2 INACTIVATION IN IRON DEFICIENCY

*Tfr2*^-/-^ mice have slightly less severe iron overload than liver-specific (*Tfr2*^LCKO^) knock-out (Wallace et al., 2007; Roetto et al., 2010), slightly higher Hb levels (Roetto et al., 2010; Nai et al., 2014) and moderate macrocytosis. The *Tmprss6*^-/-^ mice, which have a deletion of the hepcidin inhibitor, the serine protease Tmprss6, is a well established model of iron deficiency anemia with high hepcidin (Du et al., 2008; Folgueras et al., 2008). *Tmprss6*^-/-^*Tfr2* ^-/-^ double knock out animals develop iron deficiency with high hepcidin, a phenotype similar to *Tmprss6*^-/-^ mice (Lee et al., 2012; Nai et al., 2014) and to *Tmprss6*^-/-^*Hfe*^-/-^ animals (Finberg et al., 2011; Lee et al., 2012). In a single study some degree of erythrocytosis were observed both in *Tfr2*^-/-^ and in *Hfe*^-/-^ knock-out with deletion of* Tmprss6* (Lee et al., 2012), although results for *Hfe*^-/-^*Tmprss6*^-/-^ are not unequivocal (Finberg et al., 2011).

Deleting* Tmprss6* in the two hemochromatosis type 3 models, *Tfr2*^-/-^ and *Tfr2*^LCKO^ mice, revealed similarities but also differences in the hematological phenotype of the resulting double knock-out animals (Nai et al., 2014). Both models have the same degree of anemia, low transferrin saturation and low liver iron content (LIC), a phenotype quite similar to that of the iron deficient *Tmprss6*^-/-^ (**Table 1**).

The modification of the phenotype of *Tfr2*^-/-^ mice with deletion of *Tmprss6* has important implications. First it indicates that hepatic *TFR2 i*s genetically upstream *TMPRSS6* in the BMP-SMAD signaling pathway, as previously shown for *HFE* (Finberg et al., 2011; Lee et al., 2012). Second, it excludes that TFR2 is a substrate of TMPRSS6, as previously observed in our *in vitro* studies (Pagani, unpublished observation, 2014). Further, it suggests that Tmprss6 is likely hyperactive in *Tfr2*^-/-^ mice with iron overload and thus its inhibition might be effective in up-regulating hepcidin production and reducing iron overload, as shown by the use of small interference RNA (siRNA) or allele specific oligonucleotide (ASO) against *Tmprss6* (Guo et al., 2013; Schmidt et al., 2013) in *Hfe*^-/-^ animals.

**FIGURE 1:**
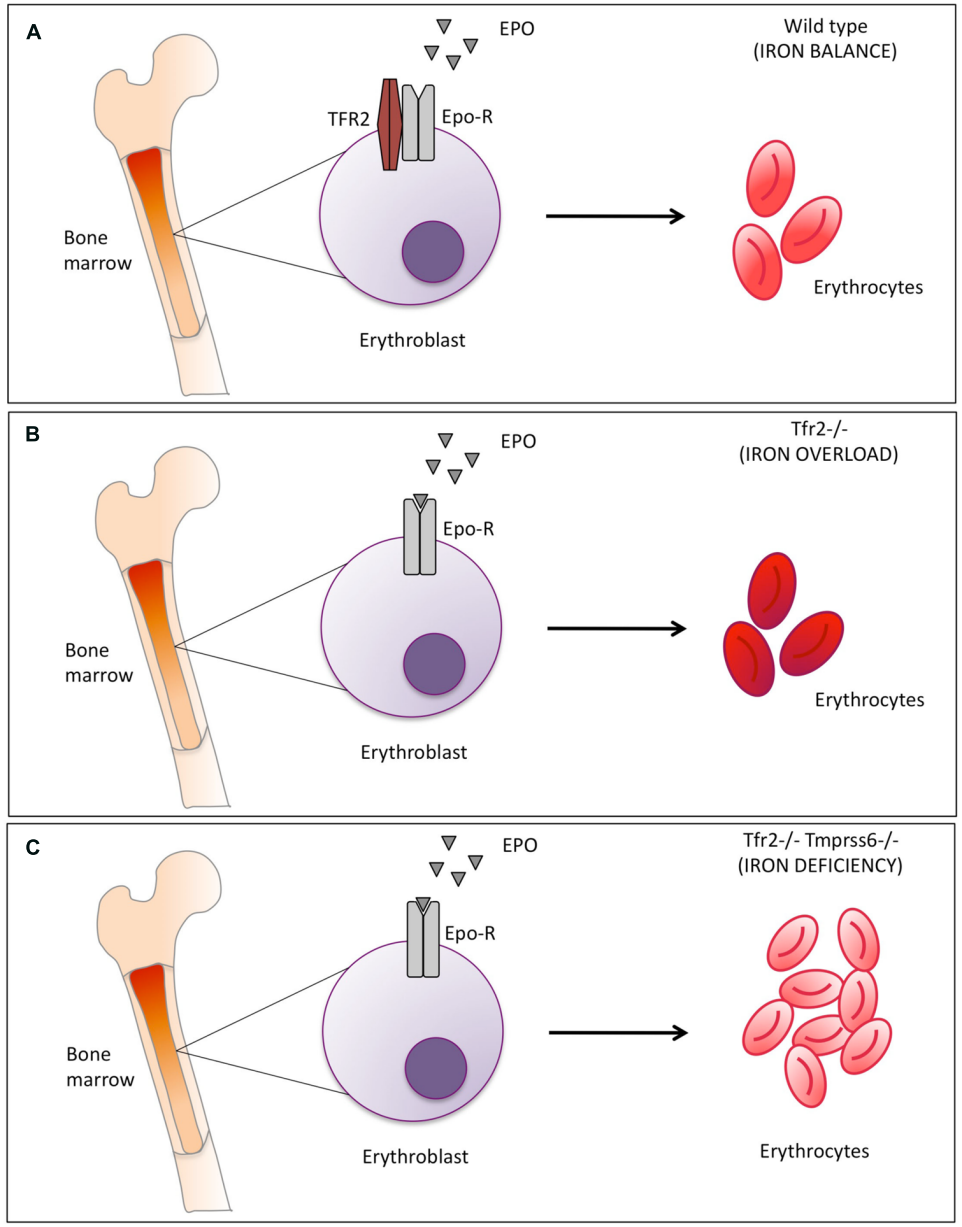
**Schematic representation of Tfr2-mediated erythropoiesis modulation. (A)** in normal conditions Tfr2 and erythropoietin receptor (EpoR) interact and localize on the cell surface and control red blood cells production **(B)** *Tfr2* inactivation in mice causes iron overload: mean corpuscular hemoglobin (MCH) is enhanced (indicated by the darker red color of the erythrocytes) due to the increased iron availability. **(C)** Inactivation of *Tmprss6* in *Tfr2* KO mice increases the number of microcytic erythrocytes, as indicated by the light red color of the erythrocytes. Epo-EpoR interaction occurs in all conditions but is increased in the absence of Tfr2 (panel B and C vs. A).

We observed that the phenotype of* Tfr2*-*Tmprss6* double knock-out mice is not exactly the same of the double knock-out for *Tfr2*^LCKO^ and *Tmprss6* or of *Tmprss6*^-/-^ knock out (**Table 1**). An increased number of red cells are observed only in *Tfr2*-*Tmprss6* double knock-out mice. Also, while hepcidin levels are increased in all models as compared with wild-type animals, they are less elevated in *Tfr2*^-/-^*Tmprss6*^-/-^ mice compared with the other models. It seems that some inhibitory signal lowers hepcidin in *Tfr2*^-/-^*Tmprss6*^-/-^ mice. In principle this may derive from the increased red cell production, exclusively present in *Tfr2*^-/-^*Tmprss6*^-/-^ mice. It is of interest that the observed erythrocytosis is not due to enhanced erythropoietin (Epo) stimulation of erythropoiesis, since Epo levels are similar and consistent with similar degrees of anemia in all models. They are even decreased in *Tmprss6*^-/-^*Tfr2*^-/-^ (Nai et al., 2014). We speculate that this positive modulation of erythropoiesis might result from the lack of *Tfr2* expression in the erythroid compartment (**Figure 1**).

Recently an erythroid function for Tfr2 was independently reported by Wallace et al. (2013) who noticed that the triple knock-out (*Tfr2*^-/-^*, Hfe*^-/-^, *Tmprss6*^-/-^) mice have more severe iron deficiency than *Tmprss6*^-/-^ mice with deletion of either *Hfe* or *Tfr2*. However, the mechanism underlining this difference remains to be worked out.

From all the data available we concluded that Tfr2 in the erythroid compartment might serve to block excessive erythropoietic expansion. If this occurs in normal conditions (**Figure 1** panel A) is difficult to verify because *Tfr2* deletion leads to iron overload. In iron overload indeed the Tfr2 erythroid function is likely masked by the excessive iron availability that increases Hb and also Hb content per single cell (**Figure 1** panel B vs. A; Roetto et al., 2010; (Nai et al., 2014). Tfr2 function becomes more evident in iron deficiency, as exemplified by *Tmprss6*^-/-^*Tfr2*^-/-^ double knock out mice (**Figure 1** panel C).

## CONCLUSION

From its identification and cloning more than 10 years ago (Kawabata et al., 1999), the correct function of TFR2 in iron metabolism has remained mysterious. Several different roles have been proposed for this receptor: originally published as a second iron importer, after the identification of *TFR2* mutations in hemochromatosis patients its proposed function became that of potential sensor of circulating iron-loaded transferrin and then of hepcidin (co)-activator. Although counteracting iron excess in the circulation remains its major function in the liver, an as well important erythropoietic function is emerging from our and other studies From the few available data it seems that TFR2 might serve as a brake to avoid iron consumption in excessive erythrocyte production in conditions of iron deficiency, likely within the perspective of global body iron economy. A prevalent role of erythroid TFR2 in iron deficiency might explain why this role is not evident in mice nor in patients with type 3 hemochromatosis, who are iron loaded and never experience iron deficiency. Further studies are needed to clarify the molecular mechanisms that mediate the TFR2 function in iron deficiency. However from now on the erythropoiesis status should be considered when interpreting the effect of TFR2 in iron metabolism and homeostasis.

## Conflict of Interest Statement

The authors declare that the research was conducted in the absence of any commercial or financial relationships that could be construed as a potential conflict of interest.
